# A double-blind, randomised, controlled Phase II trial of Heliox28 gas mixture in lung cancer patients with dyspnoea on exertion

**DOI:** 10.1038/sj.bjc.6601527

**Published:** 2004-01-20

**Authors:** S H Ahmedzai, E Laude, A Robertson, G Troy, V Vora

**Affiliations:** 1 Academic Palliative Medicine Unit, Clinical Sciences Division (South), Royal Hallamshire Hospital, Glossop Rd, Sheffield S10 2JF, UK; 2 Department of Biomedical Science, University of Sheffield, Alfred Denny Building, Western Bank, Sheffield S102TN, UK

**Keywords:** Heliox, oxygen, lung cancer, dyspnoea, exercise, 6-min walk test

## Abstract

Helium has a low density and the potential of reducing the work of breathing and improving alveolar ventilation when replacing nitrogen in air. A Phase II, double-blind, randomised, prospective, controlled trial was undertaken to assess whether Heliox28 (72% He/28% O_2_) compared with oxygen-enriched air (72% N_2_/28% O_2_) or medical air (78.9% N_2_/21.1% O_2_) could reduce dyspnoea and improve the exercise capability of patients with primary lung cancer and dyspnoea on exertion (Borg >3). A total of 12 patients (seven male, five female patients, age 53–78) breathed the test gases in randomised order via a facemask and inspiratory demand valve at rest and while performing 6-min walk tests. Pulse oximetry (SaO_2_) was recorded continuously. Respiratory rate and dyspnoea ratings (Borg and VAS) were taken before and immediately post-walk. Breathing Heliox28 at rest significantly increased SaO_2_ compared to oxygen-enriched air (96±2 cf. 94±2, *P*<0.01). When compared to medical air, breathing Heliox28 but not oxygen-enriched air gave a significant improvement in the exercise capability (*P*<0.0001), SaO_2_ (*P*<0.05) and dyspnoea scores (VAS, *P*<0.05) of lung cancer patients.

Dyspnoea is a common symptom in patients with lung malignancies with published data indicating that the prevalence is over 70% in patients in the terminal stages (Krech *et al*, 1992; Komurcu *et al*, 2000; Dudgeon *et al*, 2001a). The incidence and severity of dyspnoea increases with the progression of the disease, becoming very distressing to both patient and carers (Ahmedzai, 1998; Sergysels, 1998). Inadequate palliation of their dyspnoea has significant implications for the patient's functioning and quality of life, with resulting economic implications if potentially avoidable hospital admissions or inappropriate interventions take place.

While the mechanism of breathing and the consequences of different pathological conditions for both respiratory function and gas exchange are well known, the genesis and pathophysiology of dyspnoea as a symptom are much less well understood (Manning and Schwartzstein, 1995). There is relatively little published literature on the characterisation and management of this symptom (compared with other cancer-related conditions). Standard therapy for dyspnoea usually involves both pharmacological and nonpharmacological treatment; however, to date there has been little conclusive evidence to support the efficacy of such treatments, particularly in palliative care of patients in advanced stages (Bruera *et al* 1993; Booth *et al*, 1996; Chen *et al*, 1998; Zeppetella, 1998; Luce and Luce, 2001; Legrand, 2002,). Thus, there is an urgent need for a treatment that will reduce this common and distressing symptom.

Helium/oxygen gas mixtures have been used for many years in the management of severe upper-airway obstruction associated with tumour (Hessan *et al*, 1988; Tan *et al*, 1990), asthma (Shuie and Gluck, 1989; Kass and Castriotta, 1995; Gluck *et al*, 1990), chronic obstructive pulmonary disease (COPD)(Grape *et al*, 1960; Ishikawa and Segal, 1973; Jaber *et al*, 2000) and a variety of other conditions (Elleau *et al*, 1993; Paret *et al*, 1996; Beckmann and Brueggemann, 2000; Khanlou and Eiger, 2001). The clinical effectiveness of this respiratory gas mixture is principally due to the lower density and higher viscosity exhibited by helium/oxygen gas mixtures compared to air or oxygen alone. These properties promote a move from turbulent to more laminar flow in terminal airways and a decreased resistance to flow (Papamoschou, 1995). In selected patients, therefore, the use of Heliox28 gas mixtures may reduce the work of breathing, that is, more oxygen may be presented to the alveoli for the same ventilatory effort (DeWeese *et al*, 1983).

Clinically, helium/oxygen (Heliox) gas mixture administration is usually recommended in circumstances where a rapid decrease in respiratory muscle effort and a reduction in partial pressure of carbon dioxide (PaCO_2_) are required. This form of therapy could well provide substantial benefit for lung cancer patients with dyspnoea for whom ventilatory assistance would be inappropriate.

The principal objective of this Phase II study was to assess the efficacy of Heliox28 (helium/oxygen mixture containing 72% helium and 28% oxygen) in the palliation of dyspnoea in patients with lung cancer. Secondary objectives centred on the relative exercise capacity of patients breathing Heliox28 compared to controls of medical air and oxygen-enriched air (28% oxygen in nitrogen).

## METHODS

### Study design

This was a single-centre, double-blind, randomised, crossover study to assess the effect of Heliox28 (72% helium/28% oxygen), compared to medical air (78.9% nitrogen (N_2_)/21.1% O_2_) or oxygen-enriched air (72% N_2_/28% O_2_) in relieving dyspnoea on exertion. The study had Local Ethics (IRB) approval, and each patient gave written informed consent. Patients were recruited from lung cancer outpatient clinics after initial diagnosis and attended the study centre for an initial screening visit and a second visit not later than 2 weeks after the screening visit to assess the effects of the three gas mixtures. The study was conducted according to ICH, GCP guidelines.

### Screening visit

At this visit, all consenting patients were assessed for eligibility against the inclusion and exclusion criteria (Table 1

**Table 1:** Study conclusion/exclusion criteria

**Inclusion criteria**	**Exclusion criteria**
Male or female of age >18 years	Pregnant or lactating women
Have a diagnosis of lung cancer(radiologically and/or histologically)	Dyspnoea at rest (Borg score of 3 or more)
Dyspnoea on exertion (Borg score of 3 or more)	History of psychiatric disabilities, seizures or central nervous system disorders
Life expectancy of at least 3 monthsBe willing and able to comply with the study protocol for the duration of the study	Serious uncontrolled intercurrent infectionsHaemoglobin <10 g dl^−1^
	
	History/risk of hypercapnic respiratory failureCytotoxic chemotherapy or radiation therapy within 4 weeks of study
	
	Participation in any other investigational drug study

). Demographic data and information concerning the patient's lung cancer diagnosis were recorded (Table 2

**Table 2:** Patient Characteristics

	**Range (median)**
Age (years)	58–78 (72.5)
Transfer factor (mmol min^−1^ kPa^−1^)	2.3–8.86 (3.47)
FVC (l)	0.87–3.42 (1.94)
FEV1 (l)	0. 60–0.722 (1.26)
FEV1/FVC (%)	39–82 (72)
PEF (l min^−1^)	54–472 (185)
SaO2 at rest (%)	91–98 (94)
	
*Dyspnoea (modified Borg)*	
At rest	0–1.0 (0)
On exertion	3.0–4.0 (3.0)
	
*Pathology of lung cancer*	
Small cell	*n*=1
Non-small cell	*n*=11
	
*Stage of tumour*	
Stage I	*n*=9
Stage II	*n*=2
Stage III	*n*=1

). Standard blood haematology and biochemistry analysis was also performed. Patients were asked to rate the general extent of their dyspnoea at rest and on exertion according to both a 10 cm visual analogue scale (VAS), where 0 cm was no breathlessness and 10 cm worst breathlessness, and the modified Borg scale (Borg, 1982; Table 3

**Table 3:** Modified Borg scale

**Score**	**Symptoms**
0	Nothing at all
0.5	Very very slight (but noticeable)
1	Very slight
2	Slight
3	Moderate
4	Somewhat severe
5	Severe
6	
7	Very severe
8	
9	Very very severe
10	Maximal

). The patient's General Practitioner and other care workers were informed of the patient's participation in the study.

### Test gas mixture assessment

Vital signs, sitting blood pressure, heart rate (Blood pressure monitor, Model 711, Omron, UK), respiratory rate and tympanic temperature (Tympanic thermometer, Model 2090, IVAC Corporation, San Diego, CA, USA) were recorded. Lung transfer factor (Pulmolab, Morgan Medical, UK) was measured and the patient underwent the following spirometry assessments: forced expiratory volume in 1 s (FEV_1_), forced vital capacity (FVC) and peak expiratory flow (PEF) (MicroLab spirometer, MicroMedical Ltd, UK). Maximum inspiratory pressure (MIP) (Respiratory Muscle Trainer RT2, DeVilbiss, Sunrise Medical UK) was measured as an indirect assessment of inspiratory muscle strength and an indication of respiratory muscle fatigue.

Following these basic assessments, the patient undertook a preliminary practice 6-min walk test while breathing room air (Butland *et al*, 1982) during which the patient was encouraged in a standardised way (‘you're doing well’, or ‘keep up the good work’). This was undertaken to familiarise the patient with the procedures but did not form part of the analysis of the effects of the randomised test gas mixtures. The effects of each of the test gas mixtures on dyspnoea on exertion were then assessed during a three-period crossover phase. Helium will increase the pitch of the voice; therefore, patients were instructed not to speak while breathing the gas mixtures to avoid unblinding. Test gas mixtures were received in random sequence via a non-rebreathing mask and demand valve system (Sabre Medical Division, Sabre Safety Ltd, UK) connected via a high-pressure tubing to a portable 2 l cyclinder (BC 2 l/200 bar, BOC Ltd, Guildford, UK) carried by the investigator walking alongside the patient. Gas mixtures were administered at a rate of 8–10 l min^−1^. Distance walked was noted and pulse oximetry (SaO_2_) (Minolta PulseOx3ia, DeVilbiss, Sunrise Medical UK) recorded continuously to give resting SaO_2_ before and after breathing the gas mixture for 5 min. The minimum SaO_2_ during the 6-min walk test was also recorded.

Immediately after the 6-min walk, the patient's MIP, body temperature and respiratory rate were measured, and the extent of dyspnoea during the walk test assessed (VAS and Borg scales). A minimum 15-min rest period was given between test gas administrations.

### Statistical analysis

Results are expressed as mean±s.e.m. ANOVA was applied to the analysis of the three-period, six-sequence crossover design to explore the visual analogue data with special attention to assess potential carry-over effects. Within-patient comparisons were carried out using paired t-test with values considered significant at *P*<0.05. In addition, 95% confidence intervals (CI) are expressed for observed differences. As this was an exploratory study to determine the effect of Heliox28, for which no previous data exist in lung cancer, a formal sample size calculation for comparisons was deemed inappropriate.

## RESULTS

### Patients

In all, 12 patients were randomised and all completed the study. All 12 subjects (seven male and five female subjects) were Caucasian aged 53–78 years. Height and weight ranged from 152 to 188 cm and 47.6 to 104.8 kg, respectively. Transfer factor and spirometry values are shown in Table 2, and are indicative of mild to moderate airway obstruction. There were no clinically significant abnormal blood laboratory values.

### Walk test assessments

#### Assessment of dyspnoea (VAS and Borg scores)

**Figure 1:**
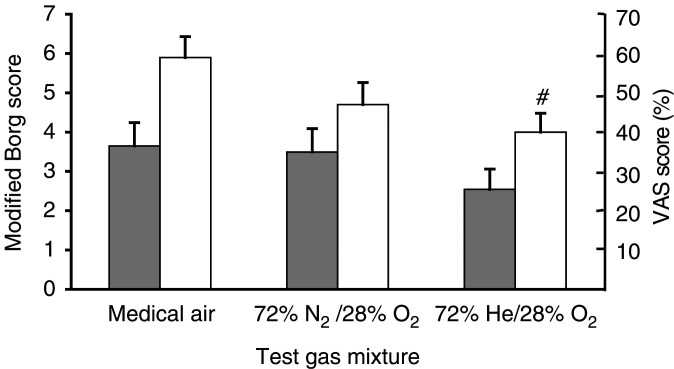
Dyspnoea scores recorded after 6-min walk test while breathing helium/oxygen (72% He/28% O_2_), oxygen-enriched air (72% N_2_/28% O_2_) and medical air (78.9% N_2_/21.1% O_2_). Mean±s.e.m. (*n*=12) modified Borg: 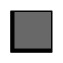; and VAS (0–10): □; #: *P*<0.01 cf. medical air.

The self-assessment of dyspnoea using the VAS showed that patients were significantly less breathless following Heliox28 (40.2±4.8%) than following medical air (59.3±5.3%) (*P*<0.05; 95% CI: 2.5–35.7%). No significant differences were detected in mean VAS between Heliox28 and oxygen-enriched air (47.0±5.6%) or between oxygen-enriched air and medical air administrations. After each walk test, modified Borg scores were 2.5±0.5 with Heliox28, 3.5±0.6 with nitrogen/oxygen and 3.7±0.6 with medical air. There were no statistically significant differences between any of the Borg score assessments (Figure 1

).

#### Distance walked

**Figure 2:**
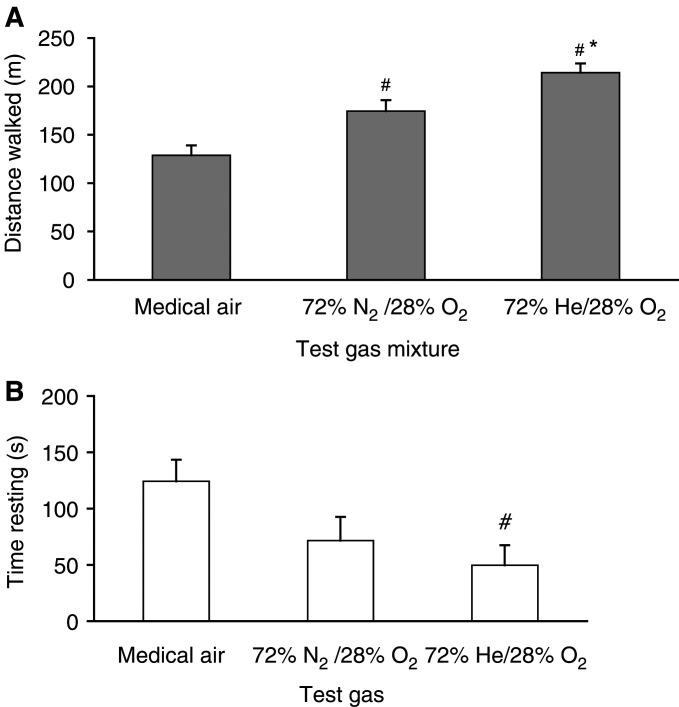
Distance walked and time spent resting during 6-min walk test while breathing test gas mixtures, helium/oxygen (72% He/28% O_2_), oxygen-enriched air (72% N_2_/28% O_2_) and medical air (78.9% N_2_/21.1% O_2_). Mean±s.e.m. (*n*=12), 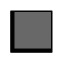: distance walked; □: time resting; #: *P*<0.01 cf. medical air; ^*^: *P*<0.05 cf. oxygen-enriched air.

Patients walked significantly further following the administration of Heliox28 (214.2±9.6 m) than following the administration of oxygen-enriched air (174.6±11.2 m) (*P*<0.05; 95% CI: 8.4–70.6 m) and medical air (128.8±10.3 m) (*P*<0.0001; 95% CI: 55.7–114.9 m). Although the distance walked with oxygen-enriched air was less than with Heliox28, it was still significantly greater than with medical air (*P*<0.01; 95% CI: 13.9–77.6 m) (Figure 2A

).

#### Time spent walking

Patients walked for longer, and spent less time resting, following the administration of Heliox28 (310±18 s) than following oxygen-enriched air (288±21 s, NS) or medical air (235±19 s; *P*<0.01; 95% CI: 19–130 s) (Figure 2B).

#### Oxygen saturation (SaO_2_)

**Figure 3:**
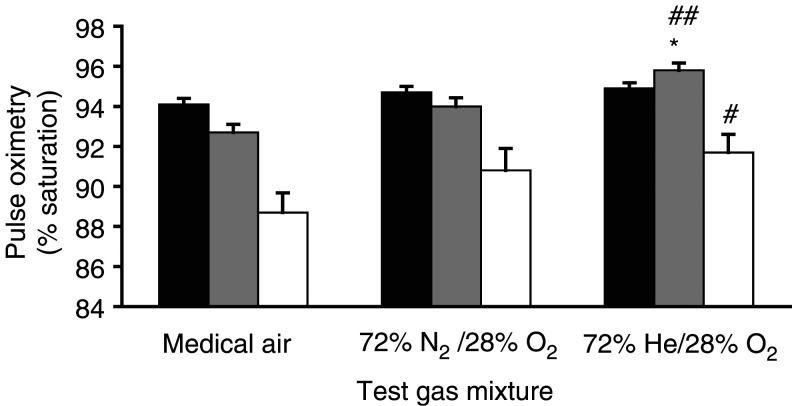
Pulse oximetry readings breathing room air, after breathing the test gas mixture at rest for 5 min and minimum reading during 6-min walk test while breathing test gases: helium/oxygen (72% He/28% O_2_), oxygen-enriched air (72% N_2_/28% O_2_) and medical air (78.9% N_2_/21.1% O_2_). ▪: initial SaO_2_; 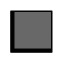: SaO_2_ after breathing gas for 5 min; □: minimum SaO_2_ during walk. Mean±s.e.m. (*n*=12) #: *P*<0.05 cf. medical air; ## : *P*<0.001 cf. medical air; ^*^: *P*<0.01 cf. oxygen-enriched air.

Initial SaO_2_ (94.9±0.28% Heliox28, 94.7±0.3% oxygen-enriched air and 94.1±0.3% medical air) on breathing room air before administration of the three test gas mixtures was not significantly different. SaO_2_ after breathing the test gas mixture for 5 min at rest before the walking test was significantly higher with Heliox28 (95.8±0.4%) than with medical air (92.7±0.4%) (*P*<0.0001; 95% CI: 1.9–4.2%) and oxygen-enriched air (94.0±0.4%) (*P*<0.01; 95% CI: 0.5–2.9%). Mean SaO_2_ value after 5 min administration of oxygen-enriched air was not significantly different from that of medical air. During the 6-min walk test, the difference between the minimum SaO_2_ recorded with the Heliox28 (91.7±0.9%) and medical air (88.7±1.0%) was statistically significant (*P*<0.05; 95% CI: 0.1–5.8%). Although the mean minimum SaO_2_ value was higher with Heliox28 than with oxygen-enriched air (90.8±1.1%), this difference was not significant (Figure 3

).

#### Maximum inspiratory pressure (MIP) and sustained MIP area

Following the baseline walk test breathing room air, the mean MIP was 35.7 cm H_2_O (range: 6–67 cm H_2_O). The MIP following each subsequent walk was 38.9±12.2, 38.2±11.8 and 36.4±10.5 cm H_2_O (Heliox28, oxygen-enriched air and medical air, respectively). There were no statistically significant treatment differences in MIP.

#### Tympanic temperature

At the end of the 5-min period breathing the test gas mixtures at rest before the walk test, mean tympanic temperature was 36.1±0.1, 36.2±0.1 and 36.3±0.1°C (Heliox28, oxygen-enriched air and medical air, respectively). Following the walk tests, there was a small but significant (*P*<0.05; 95% CI: 0.06–0.76°C) decrease in temperature after Heliox28 compared to oxygen-enriched air (35.8±0.1, 36.2±0.1 and 36.1±0.1°C, Heliox28, oxygen-enriched air and medical air, respectively).

#### Respiratory rate

After breathing the test gas mixture for 5 min at rest, respiratory rate was 18.5±0.3, 18.1±0.3 and 18.1±0.3 breaths min^−1^ (Heliox28, oxygen-enriched air and medical air, respectively). Immediately following the 6-min walks, mean respiratory rate increased to 24.2±0.6, 25.1±0.7 and 27.2±0.6 breaths min^−1^ (Heliox28, oxygen-enriched air and medical air, respectively). Compared to medical air, the exercise-induced increase was significantly lower after breathing Heliox28 (*P*<0.005; 95% CI: 1.3–4.8) and oxygen-enriched air (*P*<0.05; 95% CI: −0.2–4.0).

#### Safety

All three test gases appeared to be safe and well tolerated in this population of 12 lung cancer patients with no severe or serious adverse events or deaths reported during the study.

## DISCUSSION

This is the first randomised controlled trial of Heliox28 in patients with cancer and exertional dyspnoea. It is also the first study to use Heliox28 in this population. The results showed a consistent trend in comparisons for a therapeutic efficacy in the order of Heliox28, oxygen-enriched air and medical air gas mixtures. Dyspnoea on exercise, as assessed by VAS, was significantly lower with Heliox28 than with medical air. This proved to be a more sensitive measure in the present trial than the modified Borg score, which is used extensively in respiratory medicine studies. On average, patients walked for longer breathing Heliox28 than either medical air or oxygen-enriched air and also spent significantly less time resting than with medical air. The mean SaO_2_ after breathing the gas at rest for 5 min and minimum SaO_2_ during the walk test were both significantly higher with Heliox28 group than with oxygen-enriched air.

Breathing helium-enriched gas mixtures can reduce core body temperature because helium is a better conductor of heat than nitrogen. A slight fall in tympanic temperature was noted in our study after eleven min of continuous administration of Heliox28. This fall was statistically significant but not of a magnitude to be considered clinically significant.

There were no significant treatment differences in MIP or heart rate. As with other reports of the clinical use of Heliox28 gas mixtures (Manthous *et al*, 1997; Rodrigo *et al*, 2001), this study did not highlight any safety concerns associated with the use of Heliox28.

The results from this study support previous reports of the beneficial use of Heliox28 in the treatment of obstructive lung disease (Shuie and Gluck, 1989; Gluck *et al* 1990; Tan *et al*, 1990). All of the patients in this study were current or ex-smokers and their FEV_1_/FVC all showed varying degrees of airway obstruction but we were unable to determine how much of their obstruction was due to concomitant COPD or to their cancer. No correlation was found between the degree of airway obstruction and changes in SaO_2_, dyspnoea and distance walked in response to either Heliox28 or oxygen-enriched air.

Most of the beneficial effects of the use of inspired helium can be attributed to its physical properties of lower density and increased viscosity, compared with nitrogen. These properties lead to improved laminar gas flow in the bronchi, resulting in increased oxygenation of the alveoli and decreased carbon dioxide retention. Clinically, this has been reported in several studies as a more rapid fall in PaCO_2_ with helium/oxygen mixtures than with conventional therapy (Swidwa *et al* 1985; Oelberg *et al*, 1998; Jolliet *et al*, 1999; Kass and Terregino, 1999; Jaber *et al*, 2000). In this present study, blood gas analysis was not carried out and, therefore, PaCO_2_ values are unknown. However, the significantly higher pulse oximetry (SaO_2_) values both at rest and during exercise with Heliox28 compared to oxygen-enriched air are strongly indicative of an increase in alveolar ventilation with Heliox28.

In some COPD patients with long-standing disease, increasing their alveolar oxygenation with a high concentration of inspired oxygen raises the possibility of decreased hypoxic drive and subsequent increased carbon dioxide retention. In view of the significant degree of obstruction in this sample of patients, the BTS Guidelines (1998) were followed by using 28% oxygen in our helium mixture. Although several patients entering our study showed evidence of mild hypoxia, none could be classed as severely hypoxic (SaO_2_ <90%) or would have met the British Thoracic Society criteria for long-term oxygen therapy (SaO_2_ <92%) (BTS Guidelines, 1998).

When used to treat patients with respiratory distress, helium gas mixtures are usually administered via a system of delivery that does not allow air entrainment, thus providing the full helium/oxygen concentration to be inspired. For practical reasons, this study used a delivery system incorporating a mask and demand valve. However, any effect of the delivery system could not be quantified as the walk test breathing room air, that is without any mask, was undertaken first to familiarise the patient to the procedures and was not randomised within the test gas mixture administration.

The decreased resistance to flow of helium/oxygen mixtures may benefit subjects by decreasing the work of breathing and reducing fatigue of respiratory muscles, during periods of increased ventilatory requirement. Jaber *et al* (2000) reported a reduction in inspiration time with helium/oxygen mixtures, indicating a reduction in the work of breathing. The increases in tidal volume with helium/oxygen reported by Fink *et al* (2003), in a lung model, are interesting in this context and provide additional evidence for increased ventilation for the same ventilatory effort. MIP is often used as a measure of respiratory muscle fatigue, but in the present study between- and within-patient variability proved too great to demonstrate any significant differences. The marked weight loss found in many cancer patients with progression of the disease may also compromise ventilatory effort (Dudgeon *et al*, 2001b). We found that both Heliox28 and oxygen-enriched air enabled a reduction in the exercise-induced increase in respiratory rate in comparison to medical air, which could prove beneficial to these patients.

In general, studies of advanced cancer show that patients' perception of dyspnoea bears little direct relationship to either their SaO_2_ or lung function impairment, although there may be within-patient correlation (Bruera *et al*, 2000; Dudgeon *et al*, 2001b). Results from this study would agree with this generalisation as we could find no correlation between dyspnoea scores and lung function impairment or treatment SaO_2_ responses.

Although there is some evidence in the respiratory literature on the effectiveness of the appropriate use of oxygen in the short- and long-term management of dyspnoea in patients with chronic lung disease (Davidson *et al*, 1988; Swinburn *et al*, 1991), there is little conclusive evidence of its benefits in patients with malignancy, in particular those with lung cancer or metastatic thoracic disease.

Two randomised studies have investigated the effects of oxygen on dyspnoea in resting terminally ill cancer patients. Bruera *et al* (1993) found that 12 out of 14 patients, with hypoxaemic dyspnoea at rest, consistently preferred oxygen to air; similarly, the investigator consistently chose oxygen for the same 12 patients. In a global rating questionnaire, patients reported ‘little’ or ‘no benefit’ during the air phase compared with ‘moderate’ to ‘much benefit’ during the oxygen phase.

Booth *et al* (1996) conducted another double-blind crossover study to investigate whether oxygen aided the relief of dyspnoea at rest in 38 hospice patients with advanced cancer. Unlike Bruera's patients, their subjects were not hypoxic at rest. Mean VAS was significantly reduced following the administration of both air and oxygen, with no significant difference between the two gas mixtures.

In our study, patients were comparable to those of Booth *et al*, that is dyspnoeic on exertion but not hypoxaemic at rest. These probably represent the greater majority of cancer patients with dyspnoea. Our results indeed support those of Booth *et al* in that we could find no significant difference in relieving dyspnoea with 28% oxygen compared to medical air. However, our patients and those of Booth *et al* could not be classed as severely hypoxic at rest (SaO_2_ <90%) in contrast to those of Bruera *et al*. It is likely that the benefit from conventional oxygen therapy is greatest in those patients whose dyspnoea is a function of their low SaO_2_, as in Bruera's study. Significantly, while we were unable to identify a statistical improvement with oxygen-enriched air, Heliox28 therapy caused a significant reduction in VAS dyspnoea scores in our relatively non-hypoxic patients. The accompanying improved alveolar oxygenation and decreased ventilatory effort with Heliox28 could potentially benefit a much larger proportion of cancer patients with dyspnoea, regardless of their degree of desaturation.

The previous two cancer studies and our present one all had small sample sizes and it is possible that lack of power failed to show true significant effects. Using data from previous studies (Swidwa *et al*, 1985; Garrod *et al*, 2000), power calculations would require a group size of 22 patients to give 90% power at 5% significance for Borg scores (Machin *et al*, 1997). Our own study was designed as a Phase II trial to determine more accurately the effect size following the three test gases in the experimental situation so that future randomised trials can be designed with sufficiently large samples.

The results from this small controlled, double-blinded study suggest a promising beneficial role for Heliox28 therapy in the palliation of dyspnoea in patients with cancer. These positive clinic-based results generated interest in the more long-term benefits and we invited patients on the study to partake in a month-long home assessment. Results of this ‘real-life’ long-term monitoring will give information on functioning and quality of life in these patients, and further define the role of palliative gases in the treatment of patients with cancer-related dyspnoea.
